# Successful management of zinc phosphide poisoning—a Hungarian case

**DOI:** 10.1186/s12245-020-00307-8

**Published:** 2020-09-18

**Authors:** Gergely Bilics, Júlia Héger, Éva Pozsgai, Gábor Bajzik, Csaba Nagy, Csilla Somoskövi, Csaba Varga

**Affiliations:** 1 Department of Emergency Medicine, Somogy County Kaposi Mór General Hospital, Tallián Gyula Street 20-32, Kaposvár, 7400 Hungary; 2 Institute of Primary Health Care, Medical School, University of Pécs, Rákóczi Street 2, Pécs, 7623 Hungary; 3 Department of Public Health, Medical School, University of Pécs, Szigeti Street 12, Pécs, 7624 Hungary; 4 Department of Radiology, Somogy County Kaposi Mór General Hospital, Tallián Gyula Street 20-32, Kaposvár, 7400 Hungary; 5 Department of Neurosurgery, Clinical Center, University of Pécs, Szigeti Street 12, Pécs, 7624 Hungary; 6 Center of Psychiatry and Addictology, Somogy County Kaposi Mór General Hospital, Tallián Gyula Street 20-32, Kaposvár, 7400 Hungary; 7 Institute of Emergency Care and Pedagogy of Health, Faculty of Health Sciences, University of Pécs, Vörösmarty Mihály Street 4, Pécs, 7621 Hungary

**Keywords:** Zinc phosphide, Alpha-lipoic acid, Dihydrolipoic acid, Pneumonia, Poisoning, N-acetylcysteine, Pseudocholinesterase

## Abstract

**Background:**

Zinc phosphide (ZnP) is the basic component of several insecticides easily accessible worldwide. Intentional or accidental intoxication may lead to severe complications and multiple organ failure, resulting in high mortality. No known antidote is currently available. The iron-chelation and the antioxidative effects are well-known features of alpha-lipoic acid (ALA), although its use in the treatment of ZnP poisoning has not been documented previously. We describe the case of a patient with serious ZnP poisoning with multiple organ failure, where ALA was also included in the patient’s supportive therapy.

**Case presentation:**

A 65-year-old man ingested 125 g of Arvalin® (containing 5 g ZnP) and presented to the Emergency Department, with respiratory insufficiency and decreased consciousness. He developed hypokalemia, hypocalcemia, low white blood cell count, elevated C-reactive protein level, mixed acidosis, hepatic and kidney damage, thickening of the jejunal wall, and lung atelectasis, which served as a basis for the ensuing bacterial pneumonia. Antibiotics and adequate supportive therapy were provided. Laboratory tests indicated liver damage (slightly increased liver enzymes, low pseudocholinesterase levels; 706 U/L on day 2), possibly caused by the patient’s chronic alcoholism or the ZnP poison itself, therefore, hepatoprotective agents, ALA (Thiogamma Turbo-Set®) with N-acetylcysteine were administered for six consecutive days. Pseudocholinesterase values increased sixfold until the end of the second week of care. Fifteen days after admission, the patient was relocated to the department of psychiatry with stable vital functions, clear consciousness, declining inflammatory markers, and improved liver function. He was discharged 1 month later, fully recovered.

**Conclusions:**

Our case is the first documented voluntary and severe ZnP poisoning in Hungary. Our patient developed multiple organ failure and atelectasis, possibly resulting in the observed respiratory infection. The development of bacterial pneumonia highlighted the dangers of phosphine-induced atelectasis. The use of ALA in our patient’s case, as an antioxidant and agent for metal chelation, suggested that this agent could be a promising tool in the prevention and treatment of ZnP**-**induced hepatic damage.

## Background

Pesticides and rodenticides containing zinc phosphide (ZnP) are easily accessible chemicals worldwide. The prevalence of ZnP poisoning is comparatively higher in Asian countries, where intoxications are mainly caused by the intentional intake of the substance for suicidal purposes [1]. In contrast, cases in Europe are sporadic, and the prime reasons for poisoning are industrial accidents [2]. The ingestion of 4-5 g of ZnP is potentially lethal with a high mortality rate between 37-100% [1, 3]. The interaction between ingested ZnP and gut fluids results in the formation of phosphine gas (PH_3_) which is then absorbed through the alimentary mucosa and distributed to the body’s tissues [4]. PH_3_ damages the enzymes that play key roles in ATP synthesis, such as cytochrome c oxidase, succinate dehydrogenase, and NADH dehydrogenase [5]. PH_3_ also counteracts the antioxidant effects of the catalase and peroxidase enzymes involved in cell protective mechanisms [6]. Patients can develop a variety of symptoms, mostly gastrointestinal (68.8%), cardiovascular (22%), and respiratory symptoms (13.8%) [7]. To date, there is no known antidote for metal phosphide poisoning, although a few treatment options have been documented in case reports, such as the use of coconut oil [8], castor oil [9], tranexamic acid [10], and hemodialysis [11]. Adequate supportive therapy, often provided within an intensive care unit, is needed for recovery.

Alpha-lipoic acid (ALA) is an antioxidant and a natural coenzyme present in every cell [12] and it has been used as medicine in the treatment of diabetic and alcohol-induced liver cirrhosis [13–15]. However, the potentially beneficial effects of ALA have been studied in a broad range of other medical conditions for example in *Amanita virosa* intoxication and lead poisoning [16–18]. In our case study, we describe the case of a patient with serious ZnP poisoning, who developed multiple organ impairment and bacterial pneumonia. We also report the unique use of ALA in the supportive therapy of our patient.

## Case presentation

An ambulance was called to a 65-year-old Caucasian male with dyspnea and deteriorating consciousness. His son confirmed that the patient had ingested 125 g of Arvalin® (containing 5 g of ZnP) a few hours prior to hospital admission. The patient’s past medical history included chronic alcoholism and depression and his only regular medication was alprazolam (0.5 mg/day). Paramedics performed gastric lavage and aspired a black-colored fluid with garlic-odor from the respiratory tract, on site. Peripheral venous access was obtained and oxygen was administered through a reservoir mask due to low oxygen saturation (SpO_2_: 92%). Subsequently, the patient started wheezing and was administered 120 mg intravenously (iv.) methylprednisolone. He also vomited and since the vomit was dark-colored and upper gastrointestinal tract bleeding could not be completely excluded, 1 g tranexamic acid was given iv.

The patient was then admitted to the emergency unit with the following parameters: 142/80 mmHg blood pressure, 74/min cardiac rate, 88% SpO_2_, and a Glasgow Coma Scale of 9. Due to impaired levels of SpO_2_ and consciousness, endotracheal intubation (ETI) was initiated. The patient received assisted mechanical ventilation but his spontaneous breathing persisted (fraction of inspired oxygen 0.4; pressure support 12 cmH_2_O) and his SpO_2_ normalized. Due to agitation, the patient was sedated with midazolam (5 mg/h) then a central venous catheter was inserted into the right jugular vein.

The laboratory test showed low calcium and potassium levels, elevated C-reactive protein (CRP), and low white blood cell count (WBC). Aspartate aminotransferase (AST) and gamma-glutamyl transferase (GGT) were slightly elevated, while the patient’s alanine aminotransferase (ALT) was within the normal range. Elevated creatinine and low glomerular filtration rate (GFR) levels indicated moderate kidney impairment (Table 1).

**Table 1:** Laboratory test results of the patient upon admission to the emergency unit (day 1)

Test parameter	Reference range	Test result
White blood cells (G/L)	4.50-10.10	1.36*
Red blood cells (T/L)	4.10-5.10	4.91
Hemoglobin (g/L)	140-175	161
Hematocrite (%)	40-52	46.40
Platelet count (G/L)	100-450	169
INR	0.80-1.20	1
CRP (mg/L)	0.00-5.00	114.90*
Glucose (mmol/L)	3.30-5.50	13.50*
Sodium (mmol/L)	132-146	147*
Potassium (mmol/L)	3.70-5.40	2.76*
Calcium^2+^ (mmol/L)	1.15-1.33	0.90*
AST (U/L)	0-45	49*
ALT (U/L)	0-50	33
GGT (U/L)	8-60	57
ALP (U/L)	40-130	83
Amilase (U/L)	28-100	137*
CK (U/L)	20-200	265*
LDH (U/L)	240-480	298
Se-Bilirubin (μmol/L)	0-20	11
GFR (ml/min)	90-1000	44*
Creatinine (μmol/L)	62-106	143*
Urea (mmol/L)	0.00-11.90	11

*Numbers in bold indicate alterations from normal levels

The patient’s urine sample was positive for benzodiazepine; however, quantitative analysis was not carried out, based on the clinical symptoms.

Arterial blood gas results indicated global respiratory insufficiency, mixed acidosis with a moderately elevated anion gap (Table 2).

**Table 2:** Arterial blood gas values of the patient at the emergency unit (days 1-14)

Arterial blood gas parameters	Reference range	Day 1	Day 2	Day 5	Day 9	Day 14
Temp (C°)	35.5-37.0	37.0	36.1	36.5	37.5*	36.5
FiO_2_	0.0-1.0	0.40	0.40	0.30	0.40	0.30
pH	7.35-7.45	7.24*	7.29*	7.44	7.41	7.50*
pCO_2_(mmHg)	32-48	50.40*	56*	47.30	36.50	31.90*
pO_2_ (mmHg)	83-108	65.60*	125*	128*	88.50	70.60*
HCO_3_(mmol/L)	24+/−2	19.10*	23.60	31.20*	24	26.80*
SBE (mmol/L)	+/−2	5.10*	0.60	8*	0.80	2.10*
Lactate (mmol/L)	≤ 2	5.10*	2.10*	1.60	0.70	0.90
AGCorr (mmol/L)	16+/−4	19.20	12.90	9.70*	15.80	11.20*

*Numbers in bold indicate alterations from normal levels

**Fig. 1:**
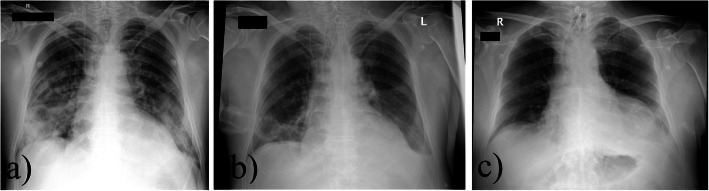
Anteroposterior chest X-rays of the patient on day 1 (**a**), 7 (**b**), 15 (**c**). **a** Day 1, confluent atelectasis on the day of admission. Central venous cannula and endotracheal tube in correct position. **b** Day 7, control examination. No signs of consolidation or atelectasis. **c** Day 15, control examination. No signs of consolidation or atelectasis. Tracheostomy tube in control position

**Fig. 2:**
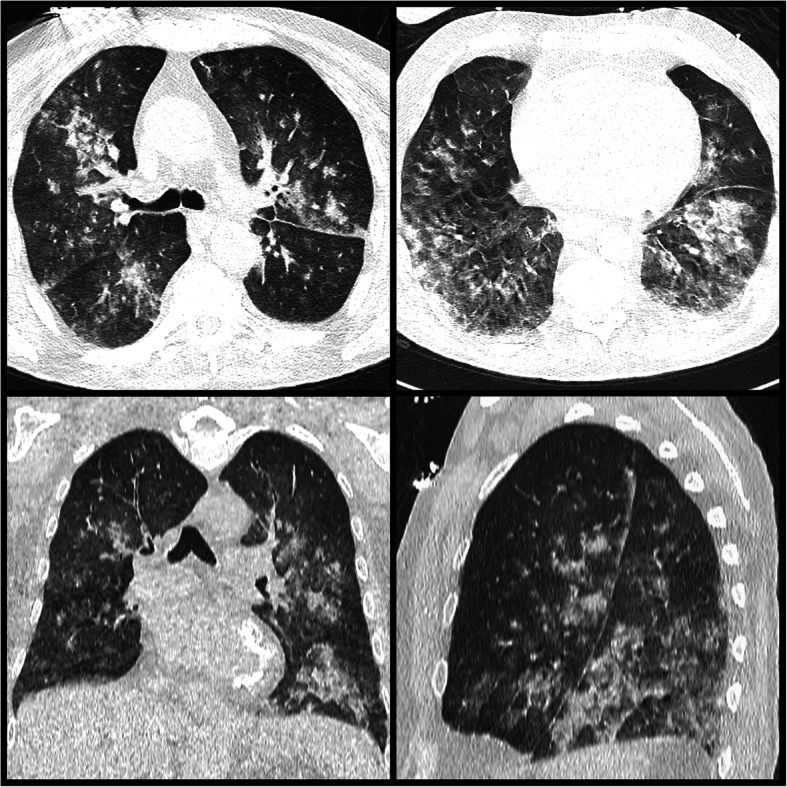
Chest CT-scan image of the patient on day 1. Axial, coronal, and sagittal plane CT images of the lung, with a 3-mm slice width made by Siemens Somatom Definition Device. Alveolar atelectasis from base to apex

Since physical examination upon admission had revealed dull percussion notes and muffled breath sounds on the right side of the thorax, as well as extensive abdominal pain, imaging was carried out. Chest X-ray and chest CT scan revealed extended alveolar infiltration of the patient’s lungs (Figs. 1a and 2).

**Fig. 3:**
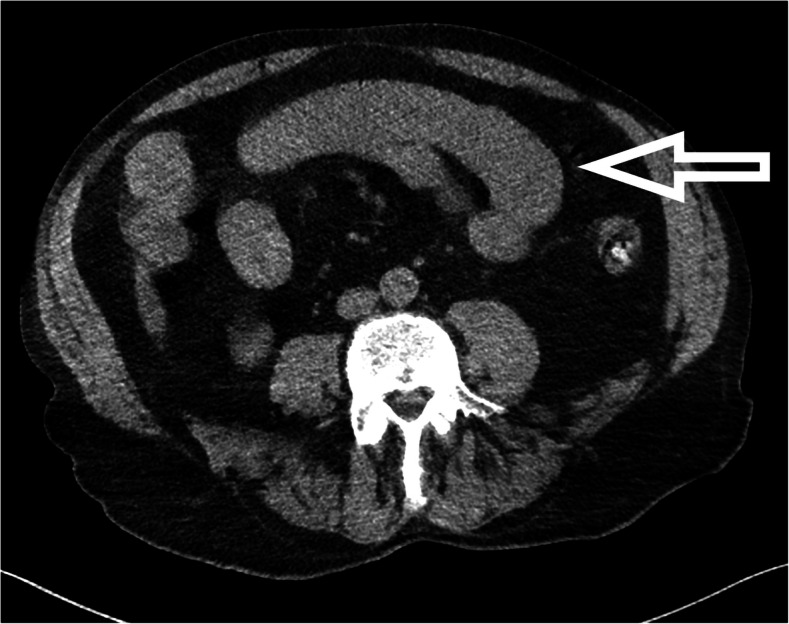
Abdominal CT-scan image of the patient on day 1. Native spiral-CT image of thorax, abdomen, and pelvis with soft tissue window and a 3-mm slice width in the axial plane. The thickened jejunal wall can be seen on the reconstructed image (marked with an arrow)

Abdominal CT scan showed the thickening of the jejunal wall (Fig. 3).

**Fig. 4:**
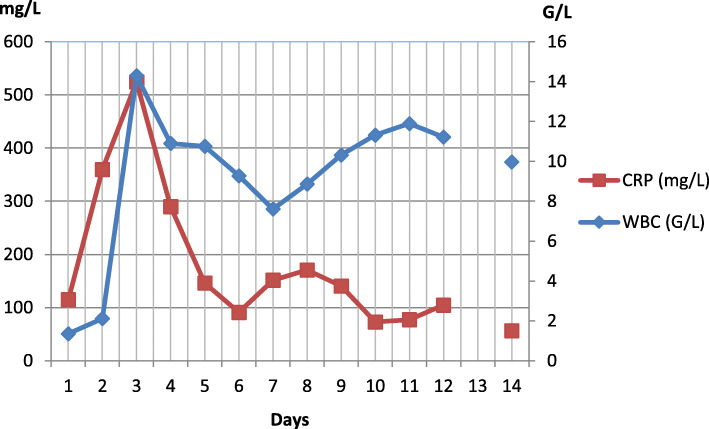
Changes of inflammatory markers (days 1-14)

Electrolyte replacement was carried out via administration of intravenous crystalloid solutions (20 mmol potassium chloride (8.4%), 1000 mg calcium gluconate in 1500 ml Isolyte® [Fresenius Kabi] iv. and 1500 ml Sterofundin B® [B. Braun Melsungen] iv.). A single dose of 30 g charcoal via nasogastric tube (NG-tube) was given. Due to the observed lung infiltration and the patient’s elevated CRP level (114.9 mg/l), ceftriaxone (2 g per day) was empirically administered and continued for 7 days (Tables 1 and 3, Fig. 4).

**Table 3:** Laboratory parameters of the patient at the emergency unit (days 1-14)

Parameter	Reference range	Day 1	Day 2	Day 3	Day 4	Day 5	Day 6	Day 7	Day 8	Day 9	Day 10	Day 11	Day 12	Day 14
**WBC (G/L)**	4.50-10.10	1.36*	2.12*	14.30*	10.90*	10.76*	9.28	7.61	8.86	10.32*	11.30*	11.90*	11.22*	9.97
**CRP (mg/L)**	0.00-5.00	114.90*	360*	525*	290*	146.10*	91.10*	151.50*	170.70*	140.60*	72.90*	77.30*	104.80*	56.40*
**ASAT (U/L)**	0-45	49*	47*	46*	28	52*	117*	44	29	24	18	18	24	24
**ALAT (U/L)**	0-50	33	31	23	19	25	65*	41	29	24	17	15	16	19
**GGT (U/L)**	8-60	57		88*				225*	194*		149*	137*	124*	
**ALP (U/L)**	40-130	83				115						79		
**ChE-PS (U/L)**	5300-13000		706*	898*		1913*	2026*							4339*
**Se-Bilirubin (μmol/L)**	0-20	11	8											
**Sodium (mmol/L)**	132-146	147*	140	134	139	147*	153*	146	150*	147*	143	146	144	143
**Potassium (mmol/L)**	3.70-5.40	2.76*	4.55	6.10*	3.90	3.79	3.39*	3.39*	4.17	4.23	3.95	3.90	3.94	3.60*
**Creatinine (μmol/L)**	62-106	143*	114*	247*	240*	159*	134*	111*	106	105	102	91	91	113*
**Urea (mmol/L)**	0.00-11.90	11	9	15*	19.40*	17.40*	13.40*	11.50	9.70	8.20	7.70	6.60	5.60	5.50
**GFR (ml/min)**	90-1000		59*	23*	23*	38*	47*	59*	63*	63*		75*		58*

*Numbers in bold indicate alterations from normal levels

Since the patient’s initial liver functions were slightly elevated (Table 1), presumably as a consequence of chronic alcohol intake, daily doses of 600 mg ALA (Thiogamma Turbo-Set®) and 2 × 600 mg N-acetylcystein (NAC) iv. were given for 6 and 7 days, respectively. Our assumption regarding the patient’s impaired liver functions was justified by the low levels of pseudocholinesterase (ChE-PS) found on the second, third, fifth, and sixth days of care (Table 3).

By the third day of the patient’s care, his potassium level had risen to 6.14 mmol/L (Table 3). He received diuretics (furosemide 6 × 10 mg and 1 × 5 mg) and glucose-insulin solution (Actrapid® 10 IE with 500 ml of Glucose B Braun® [50 mg/ml]) iv. as well as polystyrene sulfonate (1 × 50 ml) via NG-tube, whereupon his potassium level declined to a normal level. The patient’s initially moderately impaired kidney functions significantly worsened, then gradually returned to their original levels by the end of the first week (Table 3). Since the patient’s respiration became spontaneous and adequate, an attempt to remove the endotracheal tube was made. However, a few hours later tachycardia, and respiratory insufficiency occurred, therefore ETI was required again (Table 2).

On day 6, the patient developed fever and microbiological testing from blood samples was carried out. After terminating the administration of ALA, the patient’s AST and ALT levels rose slightly, then the next day, his GGT level rose substantially (Table 3).

Although still well below the normal range, the ChE-PS level had increased almost threefold (CHE-PS: 2026 U/L) by day 6 of the patient’s care, and continued to do so until day 14.

On day 7, his CRP level also began to rise (151.5 mg/L) (Table 3, Fig. 4) By the evening, the patient developed non-sustained ventricular tachycardia. Since the arrhythmia did not cause cardial instability, no specific treatment was given. No pathological signs were detected on the chest X-rays made on days 7 and 8 (Fig. 1b). Nevertheless, suspecting the possibility of a beginning respiratory infection, after consulting with an infectologist specialist, iv. ciprofloxacin (2 × 400 mg per day) was initiated.

On day 8, the patient’s blood culture was found to be positive for Acinetobacter spp. (10^4^ germ count) and sensitive to ciprofloxacin. In spite of the prevailing bacteremia and rising CRP, symptoms of sepsis did not occur, the patient’s procalcitonin level (0.25 μg/L) did not increase, and the administration of vasopressors was not required. On day 9, a second attempt was made for extubation. Due to inadequate expectoration, the patient developed dyspnea, therefore ETI was needed again. The same day a tracheostoma was inserted.

Over the next days, the patient’s hepatic enzymes and CRP level showed a slow, gradual improvement (Table 3, Fig. 4).

On day 11, trickling bleeding appeared through the tracheostoma, which ceased after the administration of tranexamic acid (1 × 1 g iv.).

The patient became agitated on day 14 therefore tiapride (1 × 100 mg iv.) and clonazepam (1 × 1 mg iv.) were given. During his psychiatric examination, the patient non-verbally verified his depression and suicidal attempt. He accepted the offered psychiatric treatment and the initiation of paroxetine (1 × 20 mg/day). Further neurological or psychiatric symptoms could not be detected.

By the end of the second week of the patient’s care, there were no radiological, and laboratory signs (WBC = 11.97G/L, CRP = 53 mg/L) of a respiratory infection (Fig. 1c). The awoken patient, with stable vital functions, was transferred to the psychiatry department of our hospital.

During his psychiatric care, oral antidepressants were added (cinalozepam 1 × 40 mg per os to paroxetine 1 × 20 mg per os) and the patient began participating in psychotherapy.

While in psychiatric care, bronchoscopy was performed, mucopurulent sputum was aspirated, and subacute mucosal inflammation was detected. The microbiological examination of the sputum revealed the presence of ciprofloxacin-sensitive Acinetobacter spp., with a germ count of 10^4^.

The patient’s clinical status gradually improved. His tracheostoma was removed on the 20th day following hospital admission and his inflammatory markers decreased to normal (WBC, 6.38 G/L) or almost normal (CRP, 5.7) levels by the 41st day of his inpatient care at the hospital. After 1 month of psychiatric inpatient care, the patient had recovered and was discharged.

## Discussion

Although there are a number of case reports and reviews about metal phosphide intoxications in Asian countries [7, 19, 20], phosphide poisonings in Europe have only been sporadically reported [2, 21]. To our knowledge, this is the first documented Hungarian case of intentional ZnP poisoning, where a particularly large dose of ZnP was ingested by the patient and yet, the patient fully recovered.

Gastric lavage is recommended for patients if phosphide intoxication took place within a few hours [9] and the use of charcoal is also advised [22], although some sources have suggested the use of potassium permanganate or coconut oil for decontamination [8, 23]. In accordance with the previous recommendations, gastric lavage and decontamination with charcoal were carried out in our patient’s case as well.

The most common symptoms of PH_3_ intoxication are abdominal pain, vomiting, dyspnea, hypotension, tachycardia, dysrhythmias, agitation, hallucination, depression, or even coma [24, 25]. The most frequently observed laboratory abnormalities are metabolic acidosis, electrolyte disturbances, leucopenia, signs of hepatic and renal failure, and anomalies in coagulation [7, 10, 26].

Our patient initially presented with abdominal pain, respiratory insufficiency, mixed acidosis with a moderately elevated anion gap, and confusion. Similarly to reports about metal phosphide poisonings, hypokalemia and hypocalcemia were also observed in our patient’s case [27, 28].

In some instances, severe ZnP poisoning can lead to cardiac failure, pulmonary edema, fulminant hepatic failure, thrombocytopenia, and disseminated intravascular coagulopathy [22, 29] and rarely, intravascular hemolysis and tubulointerstitial nephritis can also develop [30, 31]. Acute renal failure has also been reported following aluminum phosphide and ZnP intoxication [4, 31]. In line with these reports, our patient’s moderately impaired kidney functions upon admission began to deteriorate, with creatinine levels peaking on the third and fourth days. This indicated kidney damage and was probably due to the ZnP poisoning. The patient also developed respiratory insufficiency, which could have been caused by the extended atelectasis confirmed with the thoracic CT scan [22, 29]. This atelectasis could have formed the basis for the ensuing bacterial pneumonia, which developed 6 days after hospital admission. The patient’s pneumonia improved after the administration of iv. ciprofloxacin and the subsequent microbiological investigation verified the presence of an infection caused by Acinetobacter spp.

Zinc phosphide poisonings have been shown to cause typical pulmonary and abdominal complications, such as a radiopaque substance in the stomach visible with certain imaging techniques [32]. In our case, along with abdominal pain, we found the unique sign of a thickened jejunal wall on the abdominal CT scan of our patient.

Several studies have described ZnP-induced, potentially lethal, hepatic failure [33, 34]. According to Gokdemir et al., the mortality rate of patients with elevated liver enzymes after ZnP poisoning may even double [35].

Upon admission, the patient’s AST and GGT levels were slightly elevated, and the family history was positive for chronic alcoholism as well. The next day, the patient’s repeatedly low levels of ChE-PS confirmed the presence of liver injury, probably the result of ongoing alcohol consumption. ChE-PS has been shown to be a prognostic marker for liver disease. Studies have found that the level of ChE-PS was closely correlated with the damage severity of liver cells in cirrhotic patients [36]. Furthermore, determination of ChE-PS levels helped distinguish between liver disease and non-liver disease in patients, whose liver function tests (AST, ALT, GGT) were abnormal [37, 38]. The level of ChE-PS might be affected not only in liver disease but in some insecticide poisonings as well [39]. Low plasma butyrylcholinesterase activity could be used to predict the need for critical care and death in organophosphorus poisoning under certain circumstances and in an experimental study, aluminum-phosphide poisoning was shown to lead to decreased cholinesterase activity [39, 40].

Since our patient’s ChE-PS levels could have been compromised due to more than one injuring factor: alcohol-induced liver impariment and ZnP toxic injury, we initiated liver protection, and began the administration of the antioxidant ALA along with NAC. On the day of the termination of ALA treatment, the patient’s liver enzymes, AST, ALT, and GGT rose rapidly, then gradually declined to near-normal levels. An unequivocal, gradual improvement could be observed in ChE-PS levels, which increased sixfold from its initial level until the 14th day of care.

After its absorption through the alimentary mucosa, the PH_3_ formed from ZnP reaches the liver through the portal venous system. PH_3_ induces the cytoplasmic vacuolization of the hepatocytes, and causes sinusoidal congestion [41]. PH_3_ has been shown to inhibit cytochrome C oxidase, prevent peroxidase and catalase activity, cause lipid peroxidation, and disruption of the mitochondrial system and oxidative respiration [4]. ALA and its reduced form, dihydrolipoic acid have been shown to eliminate reactive oxygen species and prohibit lipid peroxidation [42]. ALA was also shown to form complexes with copper ions, thereby preventing the metal ion-induced pro-oxidative effect [43]. Former studies have proven this therapeutic effect of ALA in lead-exposed cells and rats [17, 18]. Despite these experimental studies, prior to our present patient’s case, we did not find any documented use of ALA in the treatment of metal phosphide poisoning. NAC has been shown to prevent organ toxicity including hepatotoxicity by serving as a glutathione (GSH) precursor or GSH restorer and has also proved beneficial in ZnP poisoning [34].

Two studies were carried out where patients were given a fixed-dose combination of ALA, NAC along with selenium and silymarin. These investigations found the combination to be therapeutically effective in alcoholic and viral hepatitis patients, where the significant reduction of liver function parameters was observed with no significant side effects [44, 45].

In our patient’s case, the initially decreased ChE-PS levels, indicating chronic hepatic impairment, gradually increased over the patient’s second week of care. The use of ALA in ZnP intoxication could be beneficial based on its antioxidative and chelating properties. Although the positive therapeutic effects of intensive and other forms of supportive care were most probably responsible for the successful treatment of our patient and his liver damage, we cannot exclude the possibility that the combined administration of ALA and NAC could also have been positive contributing factors. Similarly to NAC, which has been reported as a successful substance in ZnP-induced hepatotoxicity [34], there is a possibility that the addition of ALA could further enhance the prevention of the damage caused by lipid peroxidation.

Due to the known cardiotoxicity of PH_3_, the administration of magnesium sulfate, trimetazidine, or vasopressors may be required in some cases following ZnP intoxication [46–48]. During our patient’s inpatient care, non-sustained ventricular tachycardia occurred once, which neither cause cardial instability, however, nor was there a need for antiarrhythmic medication. ZnP poisoning has also been shown to cause agitation, confusion, and hallucination [49]. Since our patient was a chronic alcoholic and his confusion improved after the administration of clonazepam and tiapride, we assumed that his delirium was possibly due to alcohol withdrawal rather than to the poisoning itself.

## Conclusions

Since ZnP is the active substance of numerous, easily-accessible pesticides and can cause life-threatening complications yet ZnP poisoning is relatively less-well known, so the recognition and timely treatment of these patients is crucial. Unfortunately, there is no specific antidote that could decrease the high mortality among patients.

Our patient’s case is unique in more than one aspect. It is the first documented voluntary ZnP poisoning in Hungary. Following the ingestion of a potentially lethal dose of ZnP, multiple organs of the patient were impaired. The development of bacterial pneumonia highlighted the dangers of PH_3_-induced atelectasis, which could provide the basis for developing a severe respiratory infection. The use of ALA in our patient’s case, as an antioxidant and agent for metal chelation, suggested that this agent could contribute to the success of supportive care in the treatment of hepatic injury for metal phosphide intoxicated patients.

## Data Availability

The datasets used and/or analyzed during the current study are available from the corresponding author on reasonable request.

## Abbreviations

ZnP: Zinc phosphide
PH_3_: Phosphine gas
ALA: Alpha-lipoic acid
SpO_2_: Oxygen saturation
iv.: Intravenous
ETI: Endotracheal intubation
CRP: C-reactive protein
AST: Aspartate aminotransferase
GGT: Gamma-glutamyl transferase
ALT: Alanine aminotransferase
GFR: Glomerular filtration rate
NG: Nasogastric
NAC: N-acetylcysteine
ChE-PS: Pseudocholinesterase
